# A systematic variant annotation approach for ranking genes associated with autism spectrum disorders

**DOI:** 10.1186/s13229-016-0103-y

**Published:** 2016-10-21

**Authors:** Eric Larsen, Idan Menashe, Mark N. Ziats, Wayne Pereanu, Alan Packer, Sharmila Banerjee-Basu

**Affiliations:** 1 MindSpec Inc., 8280 Greensboro Drive, Suite 150, McLean, VA 22102 USA; 2 Department of Public Health, Faculty of Health Sciences, Ben-Gurion University of the Negev, Beer-Sheva, Israel; 3 National Institute of Child Health and Human Development, NIH, Bldg 49, Room 2c08, Bethesda, MD 20814 USA; 4 Simons Foundation Autism Research Initiative, New York, NY USA

**Keywords:** Autistic disorder, Genetic variation, Common variants, Rare variants, Autosomal recessive

## Abstract

**Background:**

The search for genetic factors underlying autism spectrum disorders (ASD) has led to the identification of hundreds of genes containing thousands of variants that differ in mode of inheritance, effect size, frequency, and function. A major challenge involves assessing the collective evidence in an unbiased, systematic manner for their functional relevance.

**Methods:**

Here, we describe a scoring algorithm for prioritization of candidate genes based on the cumulative strength of evidence for each ASD-associated variant cataloged in AutDB (also known as SFARI Gene). We retrieved data from 889 publications to generate a dataset of 2187 rare and 711 common variants distributed across 461 genes implicated in ASD. Each individual variant was manually annotated with multiple attributes extracted from the original report, followed by score assignment using a set of standardized parameters yielding a single score for each gene.

**Results:**

There was a wide variation in scores; *SHANK3*, *CHD8*, and *ADNP* had distinctly higher scores than all other genes in the dataset. Our gene scores were significantly correlated with other recently published rankings of ASD genes (*R*_Spearman_ = 0.40–0.63; *p<* 0.0001), providing support for our scoring algorithm.

**Conclusions:**

This new resource, which is freely available, for the first time aggregates on one-platform variants identified from various study types (simplex, multiplex, multigenerational, and consanguineous families), from both common and rare variants, and also incorporates their putative functional consequences to arrive at a genetically and biologically driven ranking scheme. This work represents a major step in moving from simply cataloging autism variants to using data-driven approaches to gain insight into their significance.

**Electronic supplementary material:**

The online version of this article (doi:10.1186/s13229-016-0103-y) contains supplementary material, which is available to authorized users.

## Background

Autism spectrum disorder (ASD) is a clinical diagnosis defined by neurodevelopmental impairments in two domains: persistent deficits in social reciprocity and communication across multiple contexts, together with restricted, repetitive patterns of behavior [1]. Individuals with ASD can display a broad clinical profile with varying severity in the core symptoms and often accompanied by medical comorbidities. With onset in the first years of life, ASD entails a life-long condition with diverse outcomes in adulthood [2]. The prevalence of ASD has been estimated as high as 1 in 68 children [3], yet an understanding of the biological mechanisms underlying ASD remains unclear, hampering attempts to develop specific molecular diagnostics or targeted therapeutics.

A multifactorial etiological model for ASD is being increasingly recognized. Several epidemiological studies have firmly established a genetic component underlying ASD with heritability estimates ranging from 50–90 % depending on the study parameters [4–6]. Consequently, numerous efforts to identify genes associated with ASD risk have been undertaken in hopes of inferring molecular pathways or surrogate markers associated with clinical manifestations of ASD. The ability to screen large cohorts using high-throughput genomic technologies has led to the discovery of hundreds of candidate genes containing thousands of variants, highlighting enormous genetic heterogeneity in ASD [7–10]. Although the significance of the vast majority of identified variants remains unresolved, a subset of genes have been found to be highly penetrant for ASD based on recurrent findings of rare, de novo, damaging variants in probands [11]. While initial estimates suggested between 350 and 400 autism susceptibility genes [12], more recent statistical models predict that well over 1000 genes may eventually be associated with ASD [13, 14]. Despite the incredible insight into the molecular genetics of ASD that these studies have provided, the diversity in study design, the significant variance in sample sizes and replication cohorts, and the use of different statistical models have resulted in a large set of candidate genes that are difficult to compare on a single platform. Moreover, within any given ASD candidate gene, multiple variants may be found, each with its own associated risk [15], further complicating a clear understanding of their relevance with respect to autism. To address these challenges, databases of ASD risk genes have been established in attempts to aggregate the ever-increasing number of candidate genes implicated in this disorder [16, 17]. However, only recently have strides been made towards developing methodologies for quantitative assessment of ASD risk genes [13, 18–20]. For example, transmission and de novo association (TADA) analysis was developed to identify risk-conferring genes by integrating rare de novo and inherited genetic variations from high-throughput, whole exome sequencing (WES) studies of large ASD cohorts such as the Autism Sequencing Consortium (ASC) and the Simons Simplex Collection (SSC) [11, 21]. While TADA analysis has proven to be a critical first step, further assessment strategies are required to fully integrate the complete spectrum of ASD genetic variations and consider all potential attributes that are likely to be encountered in patients evaluated in ASD clinics.

The Gene Scoring module (https://gene.sfari.org/autdb/GS_Home.do) of Simons Foundation Autism Research Initiative (SFARI) was created as a means for evaluation of candidate genes on a discrete or categorical scale taking into account the strength of genetic evidence linking a gene to ASD [22]. A set of scoring criteria was developed to assess different types of evidence, methodologies, and variability reported in the genetic studies of ASD [22]. Here, we have extended this initial work to incorporate a systematic evaluation of diverse types of genetic variants implicated in ASD. Our approach is based on assessment of multiple attributes of an ASD variant including mode of inheritance, effect size, and variant frequency in the general population. In this study, we report a consolidated gene score by summing the various evidence scores generated for each individual variant of an ASD-implicated gene. Next, we compared the gene scores generated in this study with the expert-led SFARI Gene Scoring module as well as the top ranking ASD genes identified in simplex families [11, 23]. We found strong concordance between our ASD gene ranking strategy and the other three approaches [11, 23]. Using our model, we prioritized a larger set of genes including *SHANK3*, *CHD8*, *ADNP*, *MET*, *CNTNAP2*, and others derived from the most complete collection of genetic variations associated with ASD originating from simplex, multiplex, multigenerational, and consanguineous families.

## Methods

### AutDB catalog of genetic variations associated with ASD

The AutDB database (also known as SFARI Gene) was first released in 2007 as an online portal providing a comprehensive, up-to-date resource for ASD candidate genes identified in peer-reviewed scientific publications [16]. We have since expanded the ASD gene database to include detailed annotation of both rare and common genetic variations extracted from original scientific reports. In this study, rare variants are defined as those with a population frequency <1 %; common variants are defined as those found in the general population at a frequency of ≥1 %. Annotated variants in AutDB include single nucleotide variants (SNVs), insertion-deletion variants (indels), single gene copy number variants (CNVs occurring within a gene), and chromosomal rearrangements disrupting a single gene thereby capturing the wide range of potentially pathogenic genetic variations identified in ASD cohorts. AutDB is continuously maintained by scheduled quarterly releases in order to provide the most up-to-date resource for the ASD genetics community. The data freeze of January 2015 was used for building the input dataset as described below.

### Input dataset

We performed several filtration steps to generate a dataset of ASD-specific variants to use in the scoring process (Additional file 1: Figure S1A). Annotated rare variants were excluded if they were identified in probands lacking a confirmed diagnosis of ASD (e.g., rare variants in ASD-associated genes that had been identified in patients presenting with intellectual disability or epilepsy but without ASD). Next, we excluded rare ASD-associated CNVs lacking statistical significance as determined by either the absence of a reported *p* value or a *p* value greater than 0.05. A total of 2187 rare variants remained after variant filtration. Annotated common variants were excluded from variant scoring if they were identified in cohorts lacking a confirmed diagnosis of ASD, such as common variants in ASD-associated genes found associated with other neuropsychiatric disorders such as schizophrenia and bipolar disorder. This filtration process yielded a dataset of 771 common variants associated with ASD.

### Variant scoring

Each individual variant included in the Human Gene Module of AutDB was manually annotated with 17 standardized descriptors providing a level of variant-specific detail unique to AutDB. These include significance of genetic association, family structure (simplex, multiplex, multigenerational, or consanguineous), zygosity (heterozygous, homozygous, or hemizygous), inheritance pattern (de novo or transmitted), the type of variant (missense, nonsense, etc.), and the functional effect of the variant (Additional file 2).

The functional effect of each variant represents a scoring criteria shared by both rare and common variants and is based on the reported results from the publication’s experimental analysis or in silico prediction. Then manual curation of these reported results was used to place the variant into one of four functional effect categories, with increasing weight given to variants likely to cause a functional biological effect (Additional file 3: Table S1). Variant Specificity to ASD, and Variant Inheritance and Segregation pattern of rare variants (referred to as scoring terms “R1” and “R2,” respectively) were assigned with scores reflecting the strength of the association of the variant to ASD (Additional file 3: Table S2). For instance, the R1 scoring term provides increasing weight based on the variant’s association with ASD and its absence in control populations included in the study or in large databases of human genetic variations such as dbSNP (http://www.ncbi.nlm.nih.gov/SNP/), the 1000 Genomes Project (http://www.1000genomes.org), or the NHLBI Exome Variant Server (http://evs.gs.washington.edu/EVS/). Similarly, the R2 scoring category gives increasing weight to variants exhibiting a clear segregation pattern in individuals exhibiting ASD symptoms within a pedigree. Finally, “RG5” considers the unique statistical improbability of finding multiple de novo loss-of-function (LOF) variants in a single gene in ASD cases compared to controls [9]. The “RG6” category is a similar scoring factor that adds additional weight to rare bi-allelic LOF variants identified in consanguineous families. Owing to their unique genetic architecture and contributions to ASD as compared to rare variants [4], common variants were annotated using a separate set of scoring criteria. These are summarized by the “CG1” score, which considers the reported odds ratio and associated *p* value for a variant, and the “CG2” score, which provides increasing weight if the common variant was replicated in a second cohort of ASD individuals within the same study and/or in a new study (Additional file 3: Table S3).

Finally, we developed a comprehensive algorithm that aggregates the sub-scores from each of the categories for every variant in a given gene, weights each scoring sub-category relative to each other based on their importance to ASD linkage (based on published evidence and expert opinion), and compiles a single summary score for each rare and common variant described (Additional file 1: Figure S1B and S1C, respectively). The sum of all scored rare variants and all scored common variants were then calculated to generate a total rare variant score (RVS) and/or total common variant score (CVS) for any given gene.

## Results

### ASD risk variant distributions

**Fig. 1:**
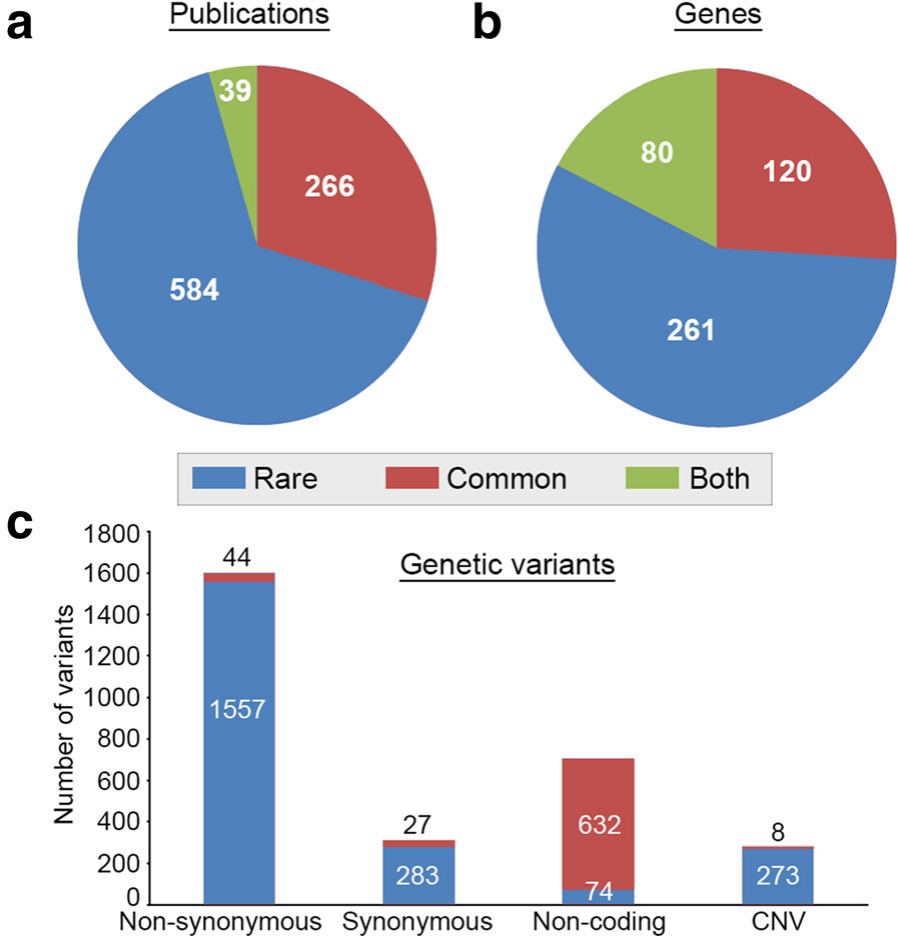
Global overview of the data in this study. **a** A pie chart showing the number of publications reporting only common (*red*), rare (*blue*), or both common and rare (*green*) genetic variants associated with autism spectrum disorder (ASD) in our database. **b** A pie chart showing the number of genes including only common (*red*), rare (*blue*), or both common and rare (*green*) genetic variants associated with ASD in our database. **c** A distribution of the common (*red*) and rare (*blue*) genetic variation in our database within four mutation categories: nonsynonymous, synonymous, non-coding, and copy number variations (CNVs)

The global landscape of variants associated with ASD analyzed in this study is summarized in Fig. 1. Overall, there were 2187 rare variants and 711 common variants in our dataset. These data were retrieved from 889 scientific publications of which 584 (65.7 %) reported only on rare variants, 266 (29.9 %) reported only on common variants, and only 39 (4.4 %) reported on both rare and common variants associated with ASD (Fig. 1a). The variants were distributed in 461 genes of which 261 (56.6 %) included only rare variants, 120 (26.0 %) included only common variants, and 80 (17.4 %) genes included both rare and common variants (Fig. 1b). Examination of the spectrum of variants in our database revealed that the majority of rare variants associated with ASD are nonsynonymous in the coding region (71.2 %), whereas most common variants are non-coding (88.9 %) (Fig. 1c).

### ASD risk genes scoring

**Fig. 2:**
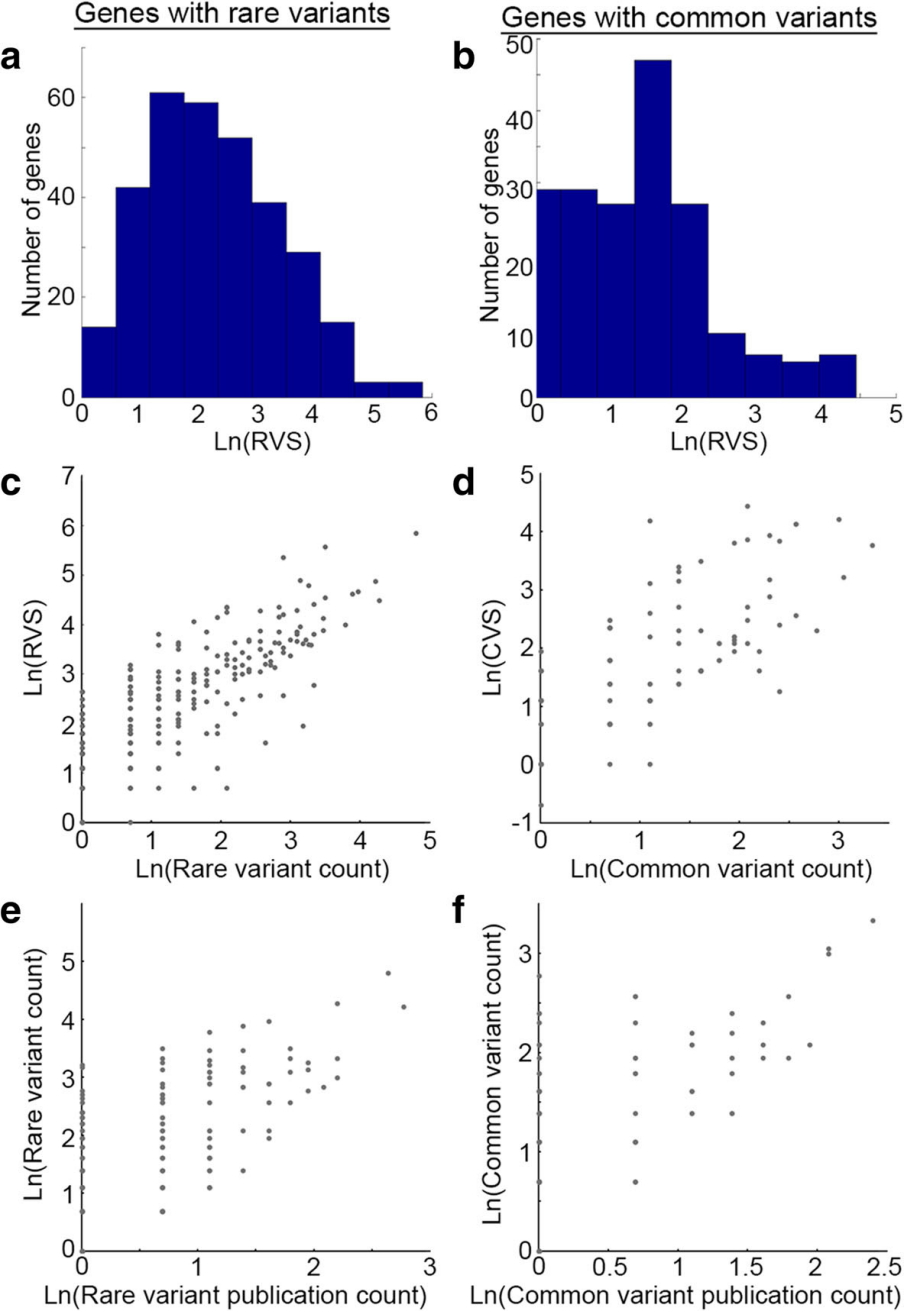
Distributions of rare variant scores (RVS) and common variant scores (CVS). The distributions of the natural logarithm of genes’ RVS and genes’ CVS are depicted as histograms (**a**, **b**, respectively), and as a function of variant counts (**c**, **d** for rare and common variants, respectively). Both RVS and CVS are significantly correlated with the number of variants per gene (*r =* 0.84 and *r =* 0.70, respectively; *p <* 0.0001). **e**, **f** The obvious association between the number of variants and the number of publication per gene (*r =* 0.70 and *r =* 0.69, respectively; *p <* 0.0001)

**Fig. 3:**
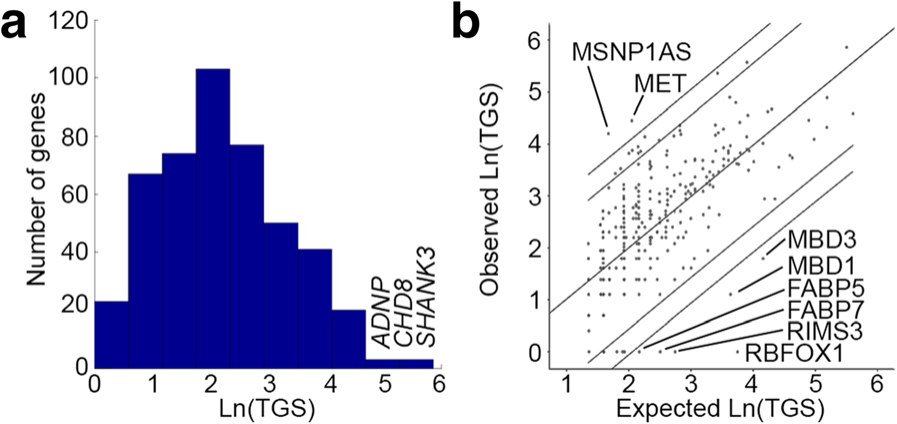
Gene scores distribution. **a** A histogram of the natural logarithm of the total gene score (LnTGS) of the 461 genes associated with autism spectrum disorder in this study. Three genes (*ADNP*, *CHD8*, and *SHANK3)* have distinctly larger scores than all other genes in the dataset. **b** A scatterplot of the expected LnTGS according to a linear regression model based on the number of variants per gene vs. the observed LnTGS. 95 and 99 % confidence intervals (CI) of the predicted scores are depicted in *dotted and dashed lines*, respectively

To assess the strength of evidence linking a candidate gene to ASD, we computed two scores for each gene: (1) rare variant score (RVS) that is based on the cumulative evidence of all rare variants within a particular gene, and (2) common variant score (CVS) which is based on the cumulative evidence of all common variants that are associated with a particular gene (Additional file 4: Table S4). Both RVSs and CVSs varied remarkably between genes (Fig. 2a, b) with *SHANK3* having the highest RVS (RVS = 346) and *MET* having the highest CVS (CVS = 85). Notably, both RVS and CVS were significantly correlated with the number of annotated variants per gene (*r =* 0.84 and *r =* 0.70, respectively; *p <* 0.0001; Fig. 2c, d), which were significantly dependent on the number of publications per gene (*r =* 0.70 and *r =* 0.69, respectively; *p <* 0.0001; Fig. 2e, f). No correlation was found between either RVS or CVS and the coding sequence length of the gene. Next, we computed a total gene score (TGS) as the sum of RVS + CVS for each gene. The TGS of all the genes in our database had a log-normal distribution with a geometric mean of TGS = 9.03 ± 3.06 (Fig. 3a). Notably, three genes (*SHANK3*, *CHD8*, and *ADNP*) had distinctively higher scores than other genes in the database.

To examine the effect of variant count on TGS, we ran a multivariate linear regression analysis using both rare and common variant counts as possible predictors. The resulting regression model explained only 50 % (adjusted *R* ^2^ = 0.502) of the variation in TGS with both rare and common variants contributing significantly to this variation (*B* = 0.83 and *B* = 0.48 for rare and common variants; *p<* 0.0001). Figure 3b displays the expected ln(TGS) scores of the genes according to this model compared to their observed ln(TGS). Six genes (*RBFOX1*, *RIMS3*, *MBD1*, *MBD3*, *FABP5*, and *FABP7*) had ln(TGS) that was significantly lower than their expected ln(TGS) according to this model (*p<* 0.01). Only two genes, *MET* and *MSNP1AS*, had ln(TGS) that were significantly higher than their expected score (*p<* 0.01).

### Comparison to other ASD risk genes datasets

**Fig. 4:**
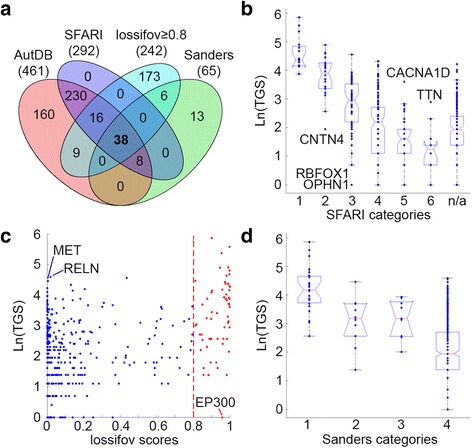
A comparison to other ASD risk genes datasets. **a** Venn diagram of the 653 ASD risk genes included in AutDB, SFARI gene scoring module [22], Iossifov et al. [23], and Sanders et al. [11]. **b** A distribution of TGS across the different categories of genes in the SFARI gene scoring module (1 = “high confidence”; 2 = “strong candidate”; 3 = “suggestive evidence”; 4 = “minimal evidence”; 5 = “hypothesized”; 6 = “not supported”). **c** A scatterplot comparing the TGS of the genes in AutDB to their corresponding posterior probabilities in Iossifov et al. [23]. Genes with posterior probabilities ≥0.8 are highlighted in *red*. **d** A distribution of TGS across the different categories of genes reported in Table 4 of Sanders et al. [11]. Categories indicate the probability pf genes to be ASD risk genes (1 = FDR ≤ 0.01; 2 = 0.01 < FDR ≤ 0.05; 3 = 0.05 < FDR ≤ 0.1; 4 = 0.1 < FDR

Next, we compared the results of our gene scoring approach to the results of three other recently published ASD-related gene sets that used other prioritization strategies. Figure 4 summarizes the results of these analyses. Of the 461 genes included in our study, 38 (8.2 %) were also included in all other gene sets and 160 (34.7 %) were exclusively represented in our AutDB (Fig. 4a). Overall, there was a strong agreement between our gene scoring approach and the other three approaches. Specifically, TGSs were significantly correlated with gene categories in the community-based SFARI gene scoring module [22] (Fig. 4b; *R*_Spearman_ = −0.63; *p<* 0.0001). Interestingly, there were few genes with TGS that deviated from the distribution of scores in their category. In addition, there were 169 genes not previously scored by the expert-led SFARI Gene Scoring initiative that were included in our analysis (Fig. 4a). These genes had a wide distribution of TGS covering almost the entire spectrum of scores in our database.

Finally, we compared our scoring results to those obtained from two recently published studies based on whole exome sequencing of ASD cohorts [11, 23]. We observed a moderate, but highly significant, correlation (*R*_Pearson_ = 0.4; *P* = 6.6 × 10^−19^) between the TGS of the 461 genes in our study and their corresponding posterior probabilities [23] (Fig. 4c). Nevertheless, only 63 (13.7 %) of these genes had a posterior probability of ≥0.8 that was suggested by Iossifov et al. [23] as good candidate ASD genes (Fig. 4a). Furthermore, of the 65 ASD risk genes reported by Sanders et al. [11], 44 (67.7 %) were included in our dataset (Fig. 4a). Here too, we observed strong correlation between the TGS of these genes and their reported ranking in that study (Fig. 4d; *R*_Spearman_ = −0.41; *p=* 4.9 × 10^−20^).

## Discussion

Given the accelerated pace of ASD candidate gene discovery, it is critical that resources be available to the research community that not only catalog the identified variants in detail but also provide tools to evaluate the potential risk conferred by each individual variant. In this report, we describe a systematic variant scoring strategy utilizing the autism gene database AutDB that encompasses detailed annotation of both rare and common genetic variants associated with ASD for candidate gene prioritization. The large set of variants analyzed here was extracted from studies that varied in size—from single case reports to analysis of large cohorts such as the Simons Simplex Collection. Additionally, our dataset included variants identified by a variety of methodologies ranging from targeted sequencing to whole genome-based screening. While a number of other recent analyses of ASD genes have focused on rare damaging de novo mutations in simplex ASD cases, our study design also allowed the inclusion of inherited autosomal recessive variants and variants observed in multiplex and multigenerational families.

This scoring approach identified three ASD risk genes (*SHANK3*, *CHD8*, and *ADNP*) that exhibited significantly higher scores than all other genes. *SHANK3* was first reported as an ASD candidate gene based on identification of heterozygous mutations in ASD probands from three unrelated families [24]. Subsequently, additional variants in *SHANK3* have been identified by targeted sequencing in multiple ASD cohorts [15, 25]. In contrast, the risk conferred by functional variants in *CHD8* and *ADNP* have only recently been described by WES studies of large ASD cohorts [9], followed by smaller studies focused exclusively on the identification of variants within these two genes [25, 26]. However, comparable WES studies of large ASD cohorts have failed to identify a large number of functional variants in *SHANK3*, due in part to the high GC content of this gene, which complicates WES approaches. These findings clearly indicate the importance of considering genetic evidence from multiple sources and multiple experimental methodologies in accurately prioritizing ASD candidate genes.

A comparison of the prioritized gene list generated by our scoring model with three other recently published ASD-related gene lists [11, 22, 23] demonstrated strong agreement in all three instances, confirming the validity of our approach. Differences in the ranking of autism candidates between our approach and these previous studies are largely due to our exclusive focus on the variant’s/gene’s role in autism, not other neurodevelopmental diseases. For example, in our approach, a candidate gene’s score is entirely dependent on the attributes of the ASD-specific genetic variants; we excluded variants from scoring when associated with a neurodevelopmental disorder without an accompanying diagnosis of ASD. By comparison, SFARI Gene Scoring takes into consideration the broader involvement of an ASD gene in related neurodevelopmental/neuropsychiatric disorders as well as its biological role in relation to ASD. These differences in scoring approaches account at least in part for the discrepancies in scores for genes such as RBFOX1 (Fig. 4b), a gene for which considerable functional evidence exists including its role in regulating other ASD genes [27, 28] and its involvement in ASD pathogenesis as manifested by differential expression in postmortem brain of ASD individuals [29]. As ASD itself is already a heterogeneous diagnosis, we built our model specifically on confirmed cases of ASD only so as to be as stringent as possible to ensure our resultant prioritization scheme is as specific to ASD as possible, as we believe this is critical to the use of such lists both for basic science researchers and especially clinicians.

An important aspect of our study is the inclusion of common variations associated with ASD (Additional file 4: Table S4). As previously indicated, *MET* had the highest common variant score (CVS = 85) based on replicated genetic association studies. Similarly, a higher evidence category was assigned to *MET* in the expert-mediated scoring in SFARI Gene. Multiple lines of research indicate an important functional role for *MET* in ASD [30]. However, the role of common variants with small effect size remains poorly understood in ASD as compared to their role in other neuropsychiatric disorders such as schizophrenia and bipolar disorder. In these other disorders, a number of common variants have reached genome-wide significance across multiple studies; common variants in ASD have by and large failed to show similar replication across independent cohorts [31, 32].

Of note is the concern that more commonly studied genes will have more variants in the database simply by virtue of having been assessed more often and therefore will rank higher in any prioritization scheme. In fact, we did find significant correlations between the total variant scores and the number of publications from which variants for a gene were extracted. This represents somewhat of a “winner’s curse” phenomenon reflecting heightened attention from the ASD research community for select genes. Nevertheless, the number of reported variants per gene (which partially reflects the scientific interest in these genes) explained only ~50 % of the variations in scores—highlighting the comprehensive nature our scoring algorithm. As more unbiased whole exome and whole genome sequencing studies are undertaken and added to this database, this effect should continue to diminish. Furthermore, ongoing future development of our algorithm will attempt to correct for such effects.

## Conclusions

In conclusion, we describe the most comprehensive database to date of both common and rare DNA variants associated with ASD. Using our novel scoring and ranking algorithm that considers both genetic and biologic data, we systematically characterized all classes of variants implicated in ASD on one platform and provide a summary score for each ASD-associated genes (Additional file 4: Table S4), which for the first time allows for a fair comparison of ASD-associated gene relevance irrespective of the type, number, or quality of study in which the underlying variant(s) were identified. In addition to strong ASD genes such as *CHD8*, *ADNP*, and *SCN2A* recurrently identified by WES, our prioritized gene set includes *SHANK3*, *MET*, and *CNTNAP2* supported by multiple lines of genetic evidence, however missed by WES. This database and ranking system represents an important step in moving from simply cataloging ASD genes to using unbiased, data-driven approaches to determine the relative strength of association with ASD of each gene. This resource, which is free to access and will continually be updated, will serve as an important tool to both basic scientists and clinicians working with ASD patients.

## Additional files

Additional file 1: Figure S1. Gene scoring process. (A) A flowchart describing the curation of both rare and common variants that are used in the overall scores of genes associated with autism spectrum disorder. (B) A formula for gene score calculation based on rare variants. (C) A formula for gene score calculation based on common variants. (TIF 1424 kb)
Additional file 2: Supplementary methods. (DOCX 12 kb)
Additional file 3: Tables S1–S3. Variant scoring criteria. Tables of scoring criteria common to both rare and common variants (Additional file 3: Table S1), scoring criteria specific for rare variants (Additional file 3: Table S2), and scoring criteria specific for common variants (Additional file 3: Table S3). (DOCX 19 kb)
Additional file 4: Table S4. Scores of ASD candidate genes using the scoring algorithm. Total gene scores were determined from the total score of all rare variants (RVS) and the total score of all common variants (CVS) for a given candidate gene. The coding sequence length of a candidate gene, the number of publications from which rare and common variants were extracted (Pub_Rare and Pub_Common, respectively), and the number of scored rare and common variants (Var_Rare and Var_Comm, respectively) are included for each gene. (XLSX 33 kb)

## Abbreviations

ASC: Autism Sequencing Consortium
ASD: Autism spectrum disorders
CNV: Copy number variant
CVS: Common variant score
LOF: Loss of function
RVS: Rare variant score
SFARI: Simons Foundation Autism Research Initiative
SNV: Single nucleotide variant
SSC: Simons Simplex Collection
TADA: Transmission and de novo association
TGS: Total gene score
WES: Whole exome sequencing
