# Supplementary figures and images for: A systematic variant annotation approach for ranking genes associated with autism spectrum disorders
Source: Mol Autism. 2016 Oct 21;7:44. doi: 10.1186/s13229-016-0103-y (PMC5075177; doi:10.1186/s13229-016-0103-y)

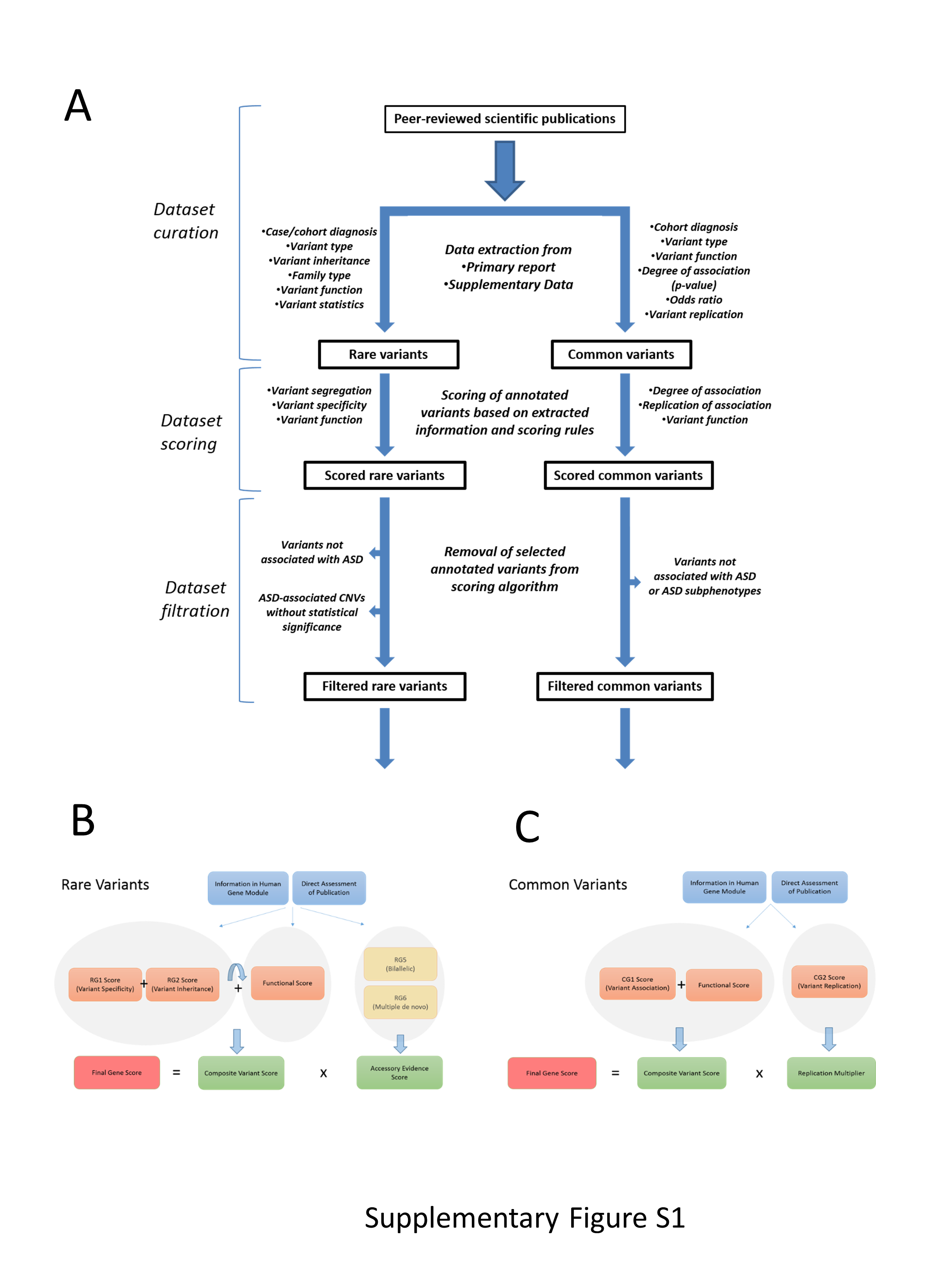

Supplement: Additional file 1: Figure S1. — Gene scoring process. (A) A flowchart describing the curation of both rare and common variants that are used in the overall scores of genes associated with autism spectrum disorder. (B) A formula for gene score calculation based on rare variants. (C) A formula for gene score calculation based on common variants. (TIF 1424 kb) [file 13229_2016_103_MOESM1_ESM.tif]
