# Supplementary material for: A systematic variant annotation approach for ranking genes associated with autism spectrum disorders
Source: Mol Autism. 2016 Oct 21;7:44. doi: 10.1186/s13229-016-0103-y (PMC5075177; doi:10.1186/s13229-016-0103-y)
Supplement: Additional file 2: — Supplementary methods. (DOCX 12 kb) [file 13229_2016_103_MOESM2_ESM.docx]

SUPPLEMENTARY METHODS

***Data Filtering***

For this analysis, we removed from the Automated Gene Scoring dataset rare variant CNVs that had not been shown to be statistically enriched in ASD cases compared to controls (i.e. those that had no accompanying p-value or had a p-value greater than 0.05). We also removed annotated variants from both the rare and common variant scoring matrices that were reported in patients whose primary diagnosis was something other than ASD using the Variant-Disorder Association criterion. This includes syndromic forms of autism such as Fragile X and Rhett Syndrome, and other neurodevelopmental disorders that often have autistic symptoms as a component such as Intellectual Disability and Dravet Syndrome. As a result of this filtering process, a total of 2187 rare variants and 711 common variants were considered in this analysis

***Computing Automated Gene Scores***

Total rare variant and total common variant scores for a given ASD-associated gene were generated by adding the scores of individual rare and/or common variants for that gene. For rare variants, the manually-annotated RG1/RG2 categories are converted to corresponding integer scores (RG1.1/RG2.1/F3.1 = 3, RG1.2/RG2.1/F3.2 = 2, RG1.3/RG2.3/F3.3 = 1 and RG1.4/RG2.4/F3.4 = 0), and then the following formula is used to obtain a Rare Variant Score for each annotated rare variant:

*Rare Variant Score = (RG1+RG2+F3)*(RG5)*(RG6)*

where RG5 and RG6 are factors that can be assigned to an individual variant to reflect the total number of biallelic loss-of-function variants or de novo loss-of-function variants, respectively, identified in a given candidate gene. All of the Rare Variant Scores for a candidate gene can then further be summed together to arrive at a final Total Rare Variant Score for that gene.

Common variants are scored in a similar matter, however owing to the unique nature of GWAS studies, our scoring model gives additional weight to common variants that demonstrate a functional significance (F3.1/3.2). In parallel to rare variants, the CG1 category annotations are converted to a corresponding integer score of 0-4, but the functional category annotations are weighted more heavily: F3.1/F3.2 = 5, F3.3 =1, and F3.4 = 0. In contrast to the rare variant formula, however, the CG2 category is used as a multiplier in the Common Variant Scoring formula:

*Common Variant score = (CG1+F3)*(CG2)*

This is done to give extra weight to variants that have been replicated in independent association studies following their initial discovery, which has been a rare occurrence in ASD and therefore deserves increased significance. The CG2 category annotations are given the following multiplier weights: CG2.1/2.2 = 4, CG2.3 = 2, CG2.4 =1. As with the rare variants, the score of each common variant for a candidate gene is summed to receive a Total Common Variant Score for that gene. Finally, the Total Gene Score for a candidate gene is determined by combining the Total Rare Variant and Total Common Variant Scores for that gene.
