# Supplementary material for: A systematic variant annotation approach for ranking genes associated with autism spectrum disorders
Source: Mol Autism. 2016 Oct 21;7:44. doi: 10.1186/s13229-016-0103-y (PMC5075177; doi:10.1186/s13229-016-0103-y)
Supplement: Additional file 3: Tables S1–S3. — Variant scoring criteria. Tables of scoring criteria common to both rare and common variants (Additional file 3: Table S1), scoring criteria specific for rare variants (Additional file 3: Table S2), and scoring criteria specific for common variants (Additional file 3: Table S3). (DOCX 19 kb) [file 13229_2016_103_MOESM3_ESM.docx]

Additional file 3

Table S1. Table of Scoring Criteria Common to Both Rare and Common Variants (Variant Function, F3)

| **Variant Function (F3)** | |
| --- | --- |
| **Category** | **Category Definition** |
| **F3.1** | Variant experimentally shown to alter cellular or neuronal processes related to ASD in vitro, iPSC-derived neurons, or other in vivo models including variant-specific animal models. |
| **F3.2** | 1. Likely gene-disruptive or loss-of-function variant based on *in silico* predictions. Such variants are typically expected to result in the generation of prematurely truncated or abnormal (i.e. out-of-frame) gene products. These variants include nonsense variants, frameshift variants, splice-site variants, or translocations/inversions with a breakpoint within the candidate gene that would be expected to disrupt the gene in question. Note: only nonsense, frameshift, and splice-site LoF variants are considered when assigning RG5 and RG6 scores.  2. Variant experimentally shown to alter gene expression, protein structure protein or function in vitro. In vitro experimental approaches are frequently used to assess functional relevance of missense variants, coding-synonymous variants, or variants in non-coding regions of a gene, or variants within intergenic regions, which may or may not be predicted to alter function based on in silico predictions. CNVs that have been experimentally shown to affect gene expression are also scored as F3.2. |
| **F3.3** | Altered variant function based on in silico predictions for missense variants and in-frame indels, or for coding-synonymous or variants in non-coding regions that are predicted to affect gene expression (i.e. binding of transcription factors) |
| **F3.4** | Variant scored as F3.4 if it meets one of the following criteria:  1. Variant shows unknown change in function (i.e., in silico predictions not performed for missense or in-frame deletion/insertion variant to assess functional importance) or no change in function (either confirmed experimentally or based on in silico predictions).  2. Variant (SNV or CNV) is located in an intergenic region (between two genes) that is not known to be a regulatory region (promoter, enchancer, etc).  3. Coding-synonymous variants, substitution variants, and CNVs within non-coding regions without predicted functional effects (such as intronic variants, 5’UTR variants or 3’UTR variants) automatically fall into this category unless they are explicitly shown (either experimentally or by in silico predictions) to have functional relevance. |
| **F3.5** | Variant scored as F3.5 if it is a deletion that contains one or more coding exons of a gene and lacks either experimental evidence showing a change in gene expression or it is not predicted to result in frameshift and premature truncation. |
| **F3.6** | Variant scored as F3.6 if it is a duplication that contains one or more coding exons of a gene and lacks either experimental evidence showing a change in gene expression or it is not predicted to result in frameshift and premature truncation. |

Table S2. Table of Scoring Criteria Specific for Rare Variants

| **Rare Variant Specificity (RG1)** | |
| --- | --- |
| **Category** | **Category Definition** |
| **RG1.1** | Variant accompanied by rigorous statistical support. Full sequencing of a comparable number of cases and controls is required. To qualify as “RG1.1”, the variant in question must fall into one of these three categories:  (1) The variant must not be present in at least two separate independent control cohorts performed in the same report  (2) The variant must not be present in an independent control cohort of matched controls (ethnically, gender, etc.) and at least one external database (EVS, 1000 Genomes, dbSNP, etc.)  (3) The variant must not be present in at least two external databases (EVS, 1000 Genomes, dbSNP, etc.). |
| **RG1.2** | Variant accompanied by strong statistical support. Full sequencing of a comparable number of cases and controls is required. To qualify as “RG1.2”, the variant in question must fall into one of these two categories:  (1) The variant must not be present in an independent control cohort of matched controls (ethnically, gender, etc.).  (2) The variant must not be present in at least 1 external database (EVS, 1000 Genomes, dbSNP, etc.) used in the report to assess specificity. |
| **RG1.3** | Variant is present in either an independent control cohort of matched controls or in an external database (EVS, 1000 Genomes, dbSNP, etc.), but still displays statistically significant enrichment in ASD cases (p<0.05). |
| **RG1.4** | A variant will be scored “RG1.4” if it fits one of the following criteria:  (1) No statistically significant difference in variant frequency between cases and an independent control cohort of matched controls (p>0.05)  (2) No statistically significant difference in variant frequency between cases and an external database (EVS, 1000 Genomes, dbSNP, etc.) (p>0.05)  (3) No information is provided in the publication to determine variant specificity (Note: this is common in case studies involving translocations and inversions). |

| **Rare Variant Inheritance and Segregation (RG2)** | |
| --- | --- |
| **Category** | **Category Definition** |
| **RG2.1** | Variant displays complete segregation with disease in a multi-generational and extended pedigree with multiple affected individuals (note: ‘disease’ refers to the broad autism phenotype and/or other neurobehavioral traits in relatives of the affected individual; minimum for ‘multigenerational’ = affected parent and affected F1 offspring) |
| **RG2.2** | Variant scored “RG2.2” if it meets one of the following criteria:  1. Variant displays complete segregation in a multiplex pedigree  Definition: a variant is transmitted from an unaffected parent only to all affected offspring (two or more) and is not present in any unaffected offspring.  2. Variant displays gender-specific or X-linked segregation with disease in a multi-generational or extended pedigree with multiple affected individuals. |
| **RG2.3** | Variant scored “RG2.3” if it meets one of the following criteria:  1. the variant arose de novo in a confirmed ASD simplex case, in ASD-affected monozygotic twins, or in a multiplex family as a result of germline mosaicism  2. the variant displays gender-specific or X-linked segregation with disease in a multiplex pedigree  3. the variant is transmitted from an unaffected parent to an affected offspring in a confirmed simplex pedigree but is not present in any unaffected offspring. |
| **RG2.4** | Unknown, incomplete, or no segregation of variant with ASD. Examples include situations where the variant is not present in all affected individuals in a multiplex pedigree, the variant is present in unaffected individuals in a simplex or multiplex pedigree, or siblings and/or parents were not sequenced in the study, even if they had ASD. |

| **RG5. Biallelic segregating LoF variants in multiplex ASD cases** | |
| --- | --- |
| ***If a candidate gene contains…*** | ***then each corresponding biallelic LoF variant will be scored as*** |
| 2 or more biallelic LoF segregating variants | **RG5.1** |
| One biallelic LoF segregating variant | **RG5.2** |
| No biallelic LoF segregating variants | **RG5.3** |

| **RG6. De novo LoF variants in simplex ASD cases** | |
| --- | --- |
| ***If a candidate gene contains…*** | ***then each corresponding de novo LOF variant will be scored as*** |
| Three or more de novo LOF variants in simplex ASD cases | **RG6.1** |
| Two de novo LOF variants in simplex ASD cases | **RG6.2** |
| One de novo LOF variant identified in a simplex ASD case | **RG6.3** |
| No de novo LOF variants | **RG6.4** |

Table S3. Table of Scoring Criteria Specific for Common Variants

| **Common Variant Association with ASD (CG1)** | |
| --- | --- |
| **Category** | **Category Definition** |
| **CG1.1** | Variant reaches genome-wide significance (GWAS; p ≤ 5.0E-08). |
| **CG1.2** | Variant does not reach genome-wide significance but shows significant association with ASD (5.0E-08 < p ≤ 1.0E-05). |
| **CG1.3** | Variant shows suggestive association with ASD (1.0E-05 < p ≤ 0.05). |
| **CG1.4** | Variant shows no association with ASD (p > 0.05). |

| **Common Variant Replication of Association (CG2)** | |
| --- | --- |
| **Category** | **Category Definition** |
| **CG2.1** | Variant scored as “CG2.1” if it showed genome-wide association with ASD in the discovery stage (CG1.1) and association with ASD was subsequently replicated in one or more independent cohorts. |
| **CG2.2** | Variant scored as “CG2.2” if it showed significant association with ASD in the discovery stage (CG1.2) and association with ASD was subsequently replicated in one or more independent cohorts. |
| **CG2.3** | Variant scored as “CG2.3” if it showed suggestive association with ASD in the discovery stage (CG1.3) and association with ASD was subsequently replicated in one or more independent cohorts. |
| **CG2.4** | Association of variant with ASD has not been replicated. |
